# Laparoscopic conversion to open surgery in radical nephrectomy and tumor thrombectomy: causal analysis, clinical characteristics, and treatment strategies

**DOI:** 10.1186/s12893-020-00845-1

**Published:** 2020-08-13

**Authors:** Zhuo Liu, Shiying Tang, Xiaojun Tian, Xun Zhao, Peng Hong, Qiming Zhang, Liwei Li, Li Zhang, Shudong Zhang, Guoliang Wang, Hongxian Zhang, Cheng Liu, Guodong Zhu, Lulin Ma

**Affiliations:** 1 Department of Urology, Peking University Third Hospital, 49 North Garden Rd, Haidian District Beijing, P.R. China; 2 Ultrasound diagnosis Department of Peking University Third Hospital, Beijing, 100083 China

**Keywords:** Laparoscopic approach, Conversion to open surgery, Renal cell carcinoma, Tumor thrombus

## Abstract

**Background:**

We aimed to explore the causal analysis, clinical characteristics and treatment strategies of laparoscopic conversion to open approach (LCTOA) in radical nephrectomy and tumor thrombectomy.

**Methods:**

We included all patients with Mayo level I–III renal tumors with inferior vena cava (IVC) tumor thrombus who underwent laparoscopic radical nephrectomy and tumor thrombectomy as the first choice from May 2015 to July 2019.

**Results:**

There were 70 cases of renal tumor with IVC tumor thrombus treated with a laparoscopic approach as the first choice; 31 Mayo level I, 30 Mayo level II, and 9 Mayo level III. A completely laparoscopic approach was performed in 51 cases (72.9%), and 19 cases (27.1%) underwent active or passive LCTOA. The LCTOA group had higher median preoperative serum creatinine (110.0 μmol/L vs 92.0 μmol/L; *P* = 0.026), longer postoperative hospital stay (9 days vs 7 days; *P* = 0.008), longer median operation time (374 min vs 311 min; *P* = 0.017), higher median intraoperative hemorrhage volume (1300 vs 600 ml; *P* = 0.020), and higher proportion of male patients (94.7% vs 66.7%; *P* = 0.016) vs the completely laparoscopic group, respectively. Although preoperative serum creatinine and gender were risk factors in the univariate analysis, multivariate analysis revealed no independent risk factors for LCTOA. We divided the reasons for LCTOA into active conversion and passive conversion; 4 (21.1%) cases underwent active conversion, and 15 (78.9%) underwent passive conversion. Most of the patients undergoing passive conversion had multiple concurrent risk factors, among which perirenal adhesion (30.9%), organ invasion (16.4%), and IVC adhesion (25.5%) were the most common. Fourteen (73.7%) cases underwent renal treatment, and 5 (26.3%) cases underwent tumor thrombus treatment.

**Conclusions:**

The LCTOA group had a higher median preoperative serum creatinine concentration, longer hospital stay, longer median operation time, and higher median intraoperative hemorrhage volume. However, none of the predictors in our study was an independent risk factor for LCTOA. Perirenal adhesion, organ invasion, and IVC adhesion were the most common causes of LCTOA. Considering the limitations of this study, studies with large sample sizes are required to validate our conclusions.

## Background

Patients with untreated renal cell carcinoma (RCC) and inferior vena cava (IVC) tumor thrombus have a poor prognosis [1, 2]. The median survival time is approximately 5 months, and the 1-year cancer specific survival rate is 29% [3]. Radical nephrectomy and tumor thrombectomy (RNATT) is a traditional and effective treatment and can effectively improve the prognosis, with a 5-year cancer specific survival rate of 40–65% [4].

Open operation is the main approach in the early stage of RCC, but this approach is associated with the disadvantages of marked trauma and long recovery time. With the popularization of the laparoscopic technique, most centers perform completely laparoscopic RNATT [5], or even robot-assisted surgery. In 1996, McDougall et al. [6] reported the first case of completely laparoscopic surgery for renal cancer with Mayo level I tumor thrombus. In 2006, Romero et al. [7] reported the first case of completely laparoscopic surgery for renal cancer with Mayo level II tumor thrombus. Laparoscopic surgery is a minimally invasive treatment with similar therapeutic effect to open surgery, but requires more involved operative technique and clinical experience. The pursuit of minimally invasive treatment should not be at the expense of therapeutic effect; and, if necessary, the minimally invasive approach should be converted to open surgery at an appropriate time.

Currently, few studies have evaluated laparoscopic conversion to an open approach (LCTOA) in RNATT. The purpose of this study was to explore the causal analysis, clinical characteristics, and treatment strategies of LCTOA in RNATT.

## Methods

### Patient selection

From May 2015 to July 2019, we enrolled all patients with Mayo level I–III renal tumors with IVC tumor thrombus undergoing laparoscopic RNATT as the first choice. The inclusion criteria were: 1) preoperative enhanced CT and/or enhanced MRI and other imaging findings showing a renal malignant tumor and IVC tumor thrombus; 2) tumor thrombus classed as Mayo level I–III; 3) laparoscopic RNATT was the first choice; and 4) renal malignant tumor confirmed as renal cell carcinoma on postoperative pathology. The exclusion criterion was that laparoscopic surgery was not the first choice for preoperative evaluation; open surgery was the first choice.

We collected the following clinical data: local symptoms (such as hematuria, lumbago, abdominal mass) or systemic symptoms (such as emaciation, fever, fatigue, anemia, etc.). Preoperatively, all patients underwent B-ultrasonographic examination to evaluate the tumor’s side, location, diameter, and relationship with the renal vessels and collecting system. TNM staging of the renal tumors was performed by chest CT scan and abdominal CT scan (UICC, 2010). Enhanced MRI was performed to measure the length of the tumor thrombus and to determine whether the tumor thrombus invaded the IVC vessel wall. The American Society of Anesthesiologists classification was used to assess the anesthesia risk [8], and renal function was evaluated according to serum creatinine concentration before and 1 week after operation [9].

### Operative method

The procedure for laparoscopic RNATT (right renal tumor) was as follows: 1) The patient was placed in the left lateral decubitus position, and the retroperitoneal cavity space was entered. 2) The right renal artery was exposed and transected. 3) The right ureter was transected after dissection. 4) Patients with right adrenal metastasis or tumor invasion underwent ipsilateral adrenalectomy. The operative procedures for the different tumor thrombus levels were as follows [10]: For Mayo level I, the IVC was exposed and partially occluded using Satinsky forceps. For Mayo level II, the distal end and proximal end of the IVC and the contralateral renal vein were exposed and occluded in sequence. First, the IVC under the renal vein (the distal end) was occluded, then the left renal vein was occluded, and finally the proximal end of the IVC was occluded. The wall of the IVC was opened, and the tumor thrombus was removed. For Mayo level III, several short hepatic veins were transected, and sufficient IVC length was exposed to provide adequate operative vision. The liver and the first porta hepatis vessels were fully exposed. First, the distal IVC was occluded, followed by the left renal vein, hepatic artery and portal vein, and finally the proximal IVC.

In patients with a left renal tumor, the retroperitoneal approach combined with the transperitoneal approach was used. Radical nephrectomy was performed by the retroperitoneal approach, and tumor thrombectomy was performed by the transperitoneal approach. We used a chevron incision for open surgery. For right RCC, the incision was located 2 cm below the right costal margin, from the xiphoid process to the axillary midline, and extending approximately 5 cm to the left of the costal margin [11].

We divided the reasons for LCTOA into active conversion and passive conversion. Active conversion meant that radical nephrectomy was performed using a completely laparoscopic approach, and tumor thrombectomy was performed by conversion to an open approach. Passive conversion meant that preoperatively, we planned to use a completely laparoscopic approach, but certain intraoperative factors necessitated conversion to an open approach.

We classified seven reasons for passive conversion: 1) Because of adhesions between the renal tumor and surrounding tissue, it was difficult to separate and expose these structures using the laparoscope. 2) The tumor invaded the surrounding tissues and organs, such as the liver, psoas major muscle, peritoneum, intestine, diaphragm, spleen, and pancreas. 3) As a result of massive bleeding in a short period during the operation, the patient’s circulatory system became unstable, such as with a decrease in blood pressure. 4) Serious adhesions between the renal vein or IVC and the surrounding tissue outside the vessel wall were found. 5) The tumor thrombus invaded the IVC endothelium. 6) After incising the IVC wall, the vessel was invaded by the tumor and needed to be removed. Additionally, the openings in the vessel wall needed to be continuously sutured. 7) Segmental resection of the IVC was performed if the tumor thrombus extensively invaded the IVC wall. If there was a long segment of loose thrombus in the distal end of the tumor thrombus, IVC transection was performed to prevent the loose section of thrombus from embolizing.

### Postoperative complications and follow-up

We used the Clavien grading system to assess and classify intraoperative and postoperative complications [12], and patients were followed-up every 6 months for 5 years, then annually thereafter. We evaluated patients’ renal function and performed abdominal B-ultrasonography and/or urinary system enhanced CT, and chest radiography or chest enhanced CT to identify local recurrence or metastasis.

### Statistical analysis

We used the Chi-square test to compare categorical variables and the Mann–Whitney U test to compare continuous variables. Univariate analysis, multivariable analysis, and Kaplan–Meier survival curves were performed using SPSS version 18 (SPSS, Inc., Chicago, IL). A *P* value ≤0.05 was considered significant.

## Results

**Fig. 1:**
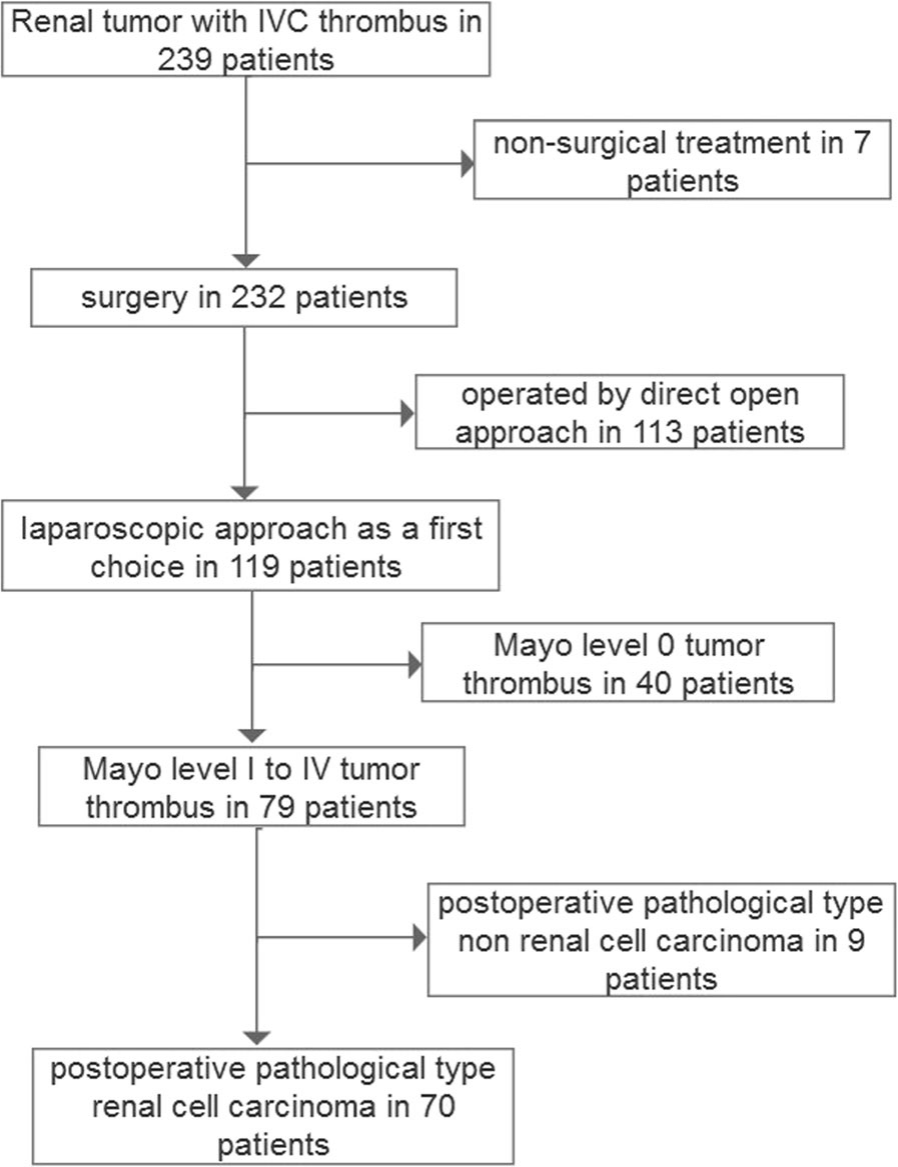
A flowchart to visualize the patient selection process

From May 2015 to July 2019, 180 cases of RCC with IVC tumor thrombus of Mayo level I–III were analyzed retrospectively in our center, namely 110 cases undergoing a completely open approach and 70 cases undergoing a laparoscopic approach as the first choice (Fig. 1). In the 70 laparoscopic cases, 51 (72.9%) cases underwent a completely laparoscopic approach, and 19 (27.1%) cases underwent LCTOA actively or passively (Table 1). The level of tumor thrombus was evaluated by Mayo grading. Among the 19 LCTOA cases, 5 cases were Mayo level I, 10 cases were Mayo level II, and 4 cases were Mayo level III.

**Table 1:** Comparison of clinical and pathologic features between complete laparoscopic surgery group and laparoscopic converse to open surgery group

	Complete laparoscopic surgery group *N* = 51 (72.9%)	Laparoscopic converse to open surgery group *N* = 19 (27.1%)	*P* value
mean value ± SD			
Age, years	61.0(51.0,68.0)	61.0(54.0,68.0)	0.995
BMI, kg/m2	22.5(20.1,27.0)	23.5(21.1,24.8)	0.958
Tumor diameter, cm	8.3(5.4,10.3)	9.1(7.1,11.0)	0.214
Albumin, g/L	39.0(37.0,42.8)	40.9(37.0,42.1)	0.672
Total Protein, g/L	69.0(66.0,74.0)	71.0(67.0,73.0)	0.376
Hemoglobin, g/L	122.0(108.0,139.0)	121.0(109.0,126.0)	0.566
Pre-operative serum creatinine, μmol/L	92.0(80.0,111.0)	110.0(89.0,130.0)	**0.026**
Serum creatinine 1 week after operation, μmol/L	103.0(86.0,117.0)	112.0(79.0,122.0)	0.658
Hospital stay after operation, days	7.0(5.0,10.0)	9.0(8.0,12.0)	**0.008**
Operative time,min	311.0(219.0,464.0)	374.0(343.0,495.0)	**0.017**
Surgical bleeding volume,ml	600.0(200.0,1500.0)	1300.0(800.0,3000.0)	**0.020**
Surgical blood transfusion volume,ml	400.0(0.0,800.0)	800.0(0.0,1600.0)	0.087
Plasma transfusion volume,ml	0.0(0.0,200.0)	0.0(0.0, 0.0)	0.699
N (%)			
Sex			
Male	34(66.7%)	18(94.7%)	**0.016**
Female	17(33.3%)	1(5.3%)	
Side			0.214
Left	16(31.4%)	9(47.4%)	
Right	35(68.0%)	10(52.6%)	
American society of anesthesiologists score			0.154
1	1(2.0%)	2(10.5%)	
2	44(86.3%)	13(68.4%)	
3	6(11.8%)	4(21.1%)	
Clinical symptoms			0.427
No clinical symptoms	14(27.5%)	2(10.5%)	
Local symptoms	25(49.0%)	10(52.6%)	
Systemic symptoms	6(11.8%)	3(15.8%)	
Both	6(11.8%)	4(21.1%)	
cN stage			0.975
cN0	19(37.3%)	7(36.8%)	
cN1	32(62.7%)	12(63.2%)	
cM stage			0.938
cM0	29(56.9%)	11(57.9%)	
cM1	22(43.1%)	8(42.1%)	
Mayo classification			0.146
I	26(51.0%)	5(26.3%)	
II	20(39.2%)	10(52.6%)	
III	5(9.8%)	4(21.1%)	
IVC resection			0.155
No	45(88.2%)	14(73.7%)	
Yes	6(11.8%)	5(26.3%)	
Pathology type			0.155
Clear cell carcinoma	45(88.2%)	14(73.7%)	
Non clear cell carcinoma	6(11.8%)	5(26.3%)	
Nuclear classification			0.856
2	16(31.4%)	5(26.3%)	
3	22(43.1%)	8(42.1%)	
4	13(25.5%)	6(31.6%)	
Rhabdoid differentiation			0.334
No	48(94.1%)	16(84.2%)	
Yes	3(5.9%)	3(15.8%)	
Sarcomatoid differentiation			1.000
No	46(90.2%)	18(94.7%)	
Yes	5(9.8%)	1(5.3%)	
Invasion of perirenal fat			0.260
No	37(72.5%)	11(57.9%)	
Yes	14(27.5%)	8(42.1%)	
Postoperative complications			0.902
No	26(51.0%)	10(52.6%)	
Yes	25(49.0%)	9(47.4%)	

The patients were classified according to the operative approach. The LCTOA group had a higher median preoperative serum creatinine (110.0 μmol/L vs 92.0 μmol/L; *P* = 0.026), longer postoperative hospitalization (9 days vs 7 days; *P* = 0.008), longer median operation time (374 min vs 311 min; *P* = 0.017), higher median intraoperative hemorrhage volume (1300 vs 600 ml; *P* = 0.020), and higher proportion of male patients (94.7% vs 66.7%; *P* = 0.016) vs the completely laparoscopic group, respectively. Although univariate analysis revealed preoperative serum creatinine and gender as risk factors, multivariate analysis showed that none of these predictors was an independent risk factor for LCTOA (Table 2).

**Table 2:** Univariate analysis and multivariate analysis for laparoscopic surgery converted to open surgery

	Univariate logistic regression analysis			Multivariate logistic regression analysis		
	OR	95%CI	P	OR	95%CI	P
Mayo classification I			0.161			0.406
Mayo classification II	2.600	0.766–8.821	0.125	1.691	0.421–6.799	0.459
Mayo classification III	4.160	0.818–21.153	0.086	3.864	0.531–28.100	0.182
cN stage	1.018	0.342–3.032	0.975	0.791	0.226–2.770	0.714
Pre-operative serum creatinine	1.027	1.001–1.054	0.039	1.014	0.987–1.042	0.316
Gender	0.111	0.014–0.904	0.040	0.168	0.017–1.630	0.124
IVC resection	2.679	0.709–10.125	0.146	2.033	0.440–9.391	0.364
Tumor diameter > 10 cm	1.400	0.461–4.247	0.552	1.228	0.329–4.579	0.760

**Fig. 2:**
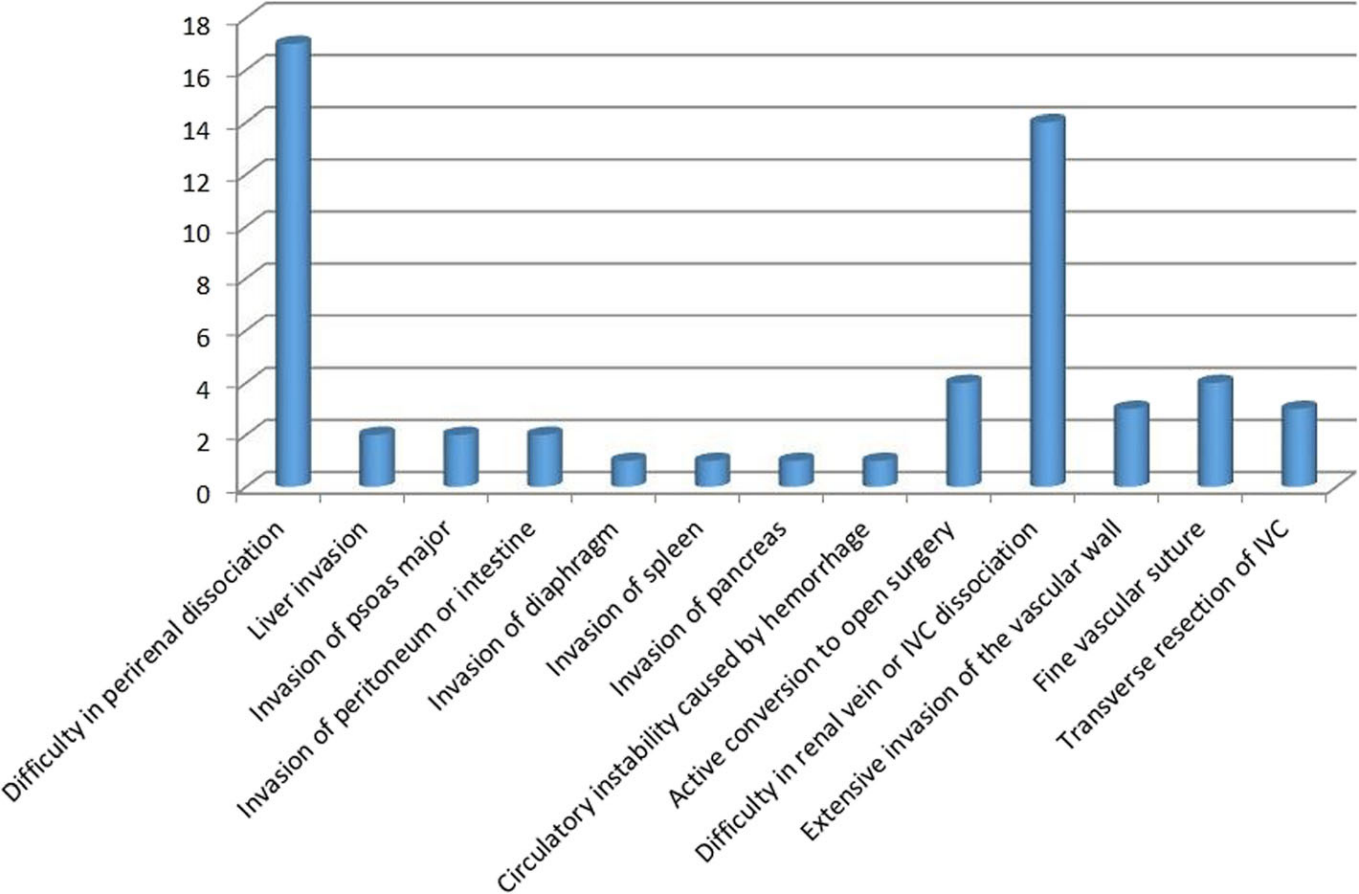
All risk factors of laparoscope conversion to open approach in radical nephrectomy and tumor thrombectomy

Four (21.1%) cases underwent active conversion, and 15 (78.9%) cases underwent passive conversion. Difficulty in perirenal dissection occurred in 17 (30.9%) patients; liver invasion occurred in 2 (3.6%) patients; psoas major invasion occurred in 2 (3.6%) patients; peritoneal or intestinal invasion occurred in 2 (3.6%) patients; diaphragmatic invasion occurred in 1 (1.8%) patient; splenic invasion occurred in 1 (1.8%) patient; pancreatic invasion occurred in 1 (1.8%) patient; circulatory instability caused by hemorrhage occurred in 1 (1.8%) patient; difficulty in renal vein or IVC dissection occurred in 14 (25.5%) patients; extensive invasion of the vascular wall occurred in 3 (5.5%) patients; precise vascular suturing was required in 4 (7.3%) patients; and transverse IVC resection occurred in 3 (5.5%) patients (Fig. 2).

**Fig. 3:**
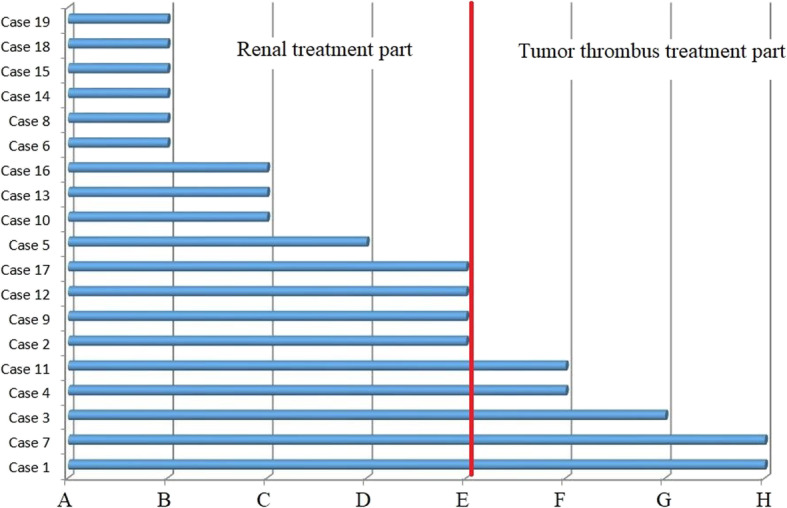
Laparoscope conversion to open approach in renal treatment part and tumor thrombus treatment part. In renal treatment part: 1) From A to B: The kidney was dissociated along the perirenal fascia; 2) From B to C: To separate the anatomical layers between the renal tumor and the surrounding tissues and organs; 3) From C to D: circulatory instability caused by hemorrhage; 4) From D to E: active conversion to open surgery. In tumor thrombus treatment part: 1) From E to F: To separate the anatomical layers between IVC and surrounding tissues and organs; 2) From F to G: The IVC tumor thrombus was removed; 3) From G to H: The walls of IVC were sutured

According to the different operation areas, the operation steps were divided into two categories: renal treatment and tumor thrombus treatment. In our center, we usually gave priority to renal treatment and then performed tumor thrombus treatment. Renal treatment was as follows: 1) The kidney was dissected along the perirenal fascia (Fig. 3 from A to B), and LCTOA was performed in 6 (31.6%) patients. 2) The right renal artery was exposed and transected. 3) The anatomical layers between the renal tumor and the surrounding tissues and organs were dissected and separated (Fig. 3 from B to C). LCTOA was performed in 3 (15.8%) patients, and circulatory instability caused by hemorrhage (Fig. 3 from C to D) occurred in 1 (5.3%) patient. During tumor thrombus treatment, active conversion to open surgery (Fig. 3 from D to E) occurred in 4 (21.1%) patients. 4) The anatomical layers between the IVC and surrounding tissues and organs were separated (Fig. 3 from E to F). LCTOA was performed in 2 (10.5%) patients. 5) The corresponding vessels were occluded. 6) The walls of the IVC were incised. 7) The IVC tumor thrombus was removed (Fig. 3 from F to G). LCTOA was performed in 1 (5.3%) patient. 8) The involved vessel wall was removed. 9) The walls of the IVC were sutured (Fig. 3 from G to H), and LCTOA was performed in 2 (10.5%) patients. 10) Occlusion in the corresponding vessels was released. Fourteen (73.7%) cases underwent renal treatment, and 5 (26.3%) cases underwent tumor thrombus treatment (Fig. 3).

Of the 19 patients in the LCTOA group, 9 patients had postoperative complications. One case developed a grade I complication (wound infection). Six patients developed grade II complication; 3 patients developed lower extremity venous thrombosis, 2 patients required blood transfusions because of postoperative anemia, and 1 patient developed atrial fibrillation. Two patients developed grade IVa complications; both developed renal insufficiency.

Of the 51 patients in the complete laparoscopic approach group, 9 patients developed postoperative complications. Five patients developed grade II complications; 3 cases of postoperative pneumonia, 1 case of atrial fibrillation, and 1 case of postoperative intestinal obstruction. Three patients developed grade IVa complication; all three developed renal insufficiency. One patient developed a grade IVa complication of acute cerebral infarction with bilateral lower extremity venous thrombosis.

**Fig. 4:**
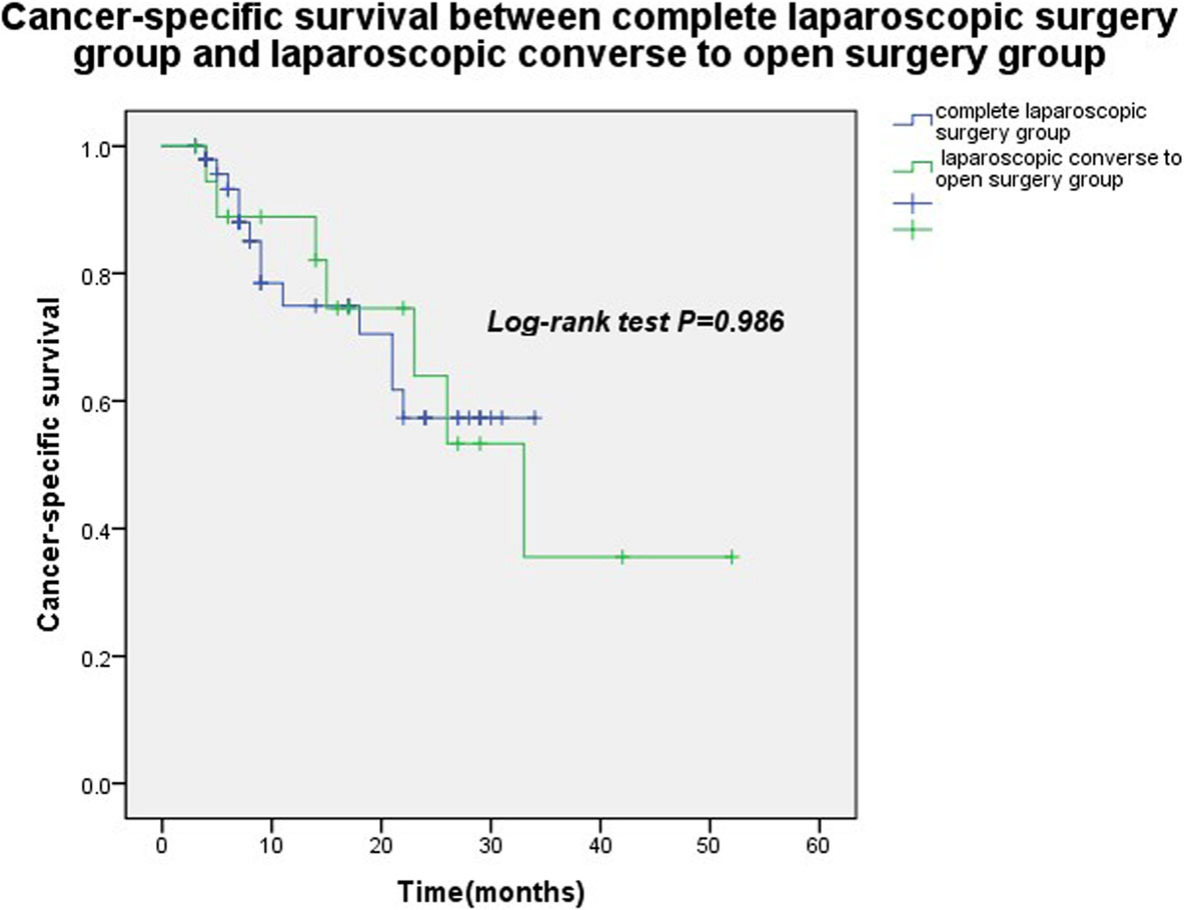
Cancer-specific survival time between laparoscope conversion to open approach (LCTOA) group and complete laparoscopic approach group

The median follow-up time was 10 months (range: 3–52 months). The mean cancer-specific survival time in the completely laparoscopic approach group was 25.0 ± 2.0 months, while that of the LCTOA group was 32.1 ± 5.1 months (*P* = 0.986). There was no statistical difference in survival between the groups. LCTOA achieved the same tumor control effect as the completely laparoscopic approach group (Fig. 4).

## Discussion

Compared with the completely laparoscopic approach group, the LCTOA group had longer operation time, increased intraoperative hemorrhage volume, and longer postoperative hospitalization duration, which may be related to the larger degree of trauma with the open approach [13]. However, open surgery remains a traditional and safe treatment strategy. Regarding the risk factors for LCTOA, we found no independent preoperative predictors in the multivariable analysis. Clinically, according to our experience, surgeons should be well prepared for LCTOA for the following patients: 1) Preoperative imaging examination shows that the tumor has invaded the surrounding tissues and organs. 2) The patient has severe perirenal adhesions. When evaluating perirenal adhesions, the Mayo Adhesive Probability Score could help judge the degree of perirenal adhesions [14]. 3) Patients with a tumor that has extensively invaded the blood vessel wall; and preoperative abdominal MRI indicates a rough IVC wall, obviously thickened IVC, or the lateral IVC wall is obviously remodeled.

Regarding the LCTOA classifications, the proportion of active conversions was 21.1%. Active conversion combined the advantages of minimally invasive treatment using the laparoscopic approach with the safety of an open approach. Additionally, the retroperitoneal laparoscopic approach has advantages regarding ligating the renal artery. During tumor thrombus treatment, the open approach has the advantages of a larger operation space and easier bleeding control. Compared with active conversion, the incidence of passive conversion was higher (78.9%). Passive conversion required the ability of timely response, confident decision-making ability, and good doctor–patient communication. Most patients undergoing passive conversion have multiple concurrent risk factors, most commonly perirenal adhesions, organ invasion, and IVC adhesion. Therefore, if it becomes difficult to complete the operation laparoscopically, the operation should be immediately and decisively converted to open surgery.

In our study, of the 19 patients in the LCTOA group, 9 patients had postoperative complications, and 2 patients had severe (Clavien grade ≥ 3) complications. In previous studies, we constructed an accurate preoperative model to predict overall postoperative complications in patients with renal cell carcinoma and tumor thrombus [15]. Conversion to open surgery itself could be considered an intraoperative complication. Regarding the timing of LCTOA, 73.7% underwent LCTOA during renal treatment, and 26.3% underwent LCTOA during tumor thrombus treatment, which differed from our previous hypothesis. We previously thought that the complexity of tumor thrombus surgery was mainly reflected in the process of tumor thrombectomy, but successful nephrectomy was also very important, and nephrectomy should receive greater attention. Another reason for the difference between our hypothesis and results is that some medical centers considered that “IVC first” was necessary to reduce the risk of a loose tumor thrombus embolizing intraoperatively. However, in our center, we usually gave priority to radical nephrectomy for broader operation space and better control of blood vessels.

Centralization of care for complex surgeries, such as radical nephrectomy with IVC thrombectomy, was also considered to influence the intra- and postoperative results. High-volume hospitals usually have better centralization of care, such as the presence of intensive care units, multi-disciplinary treatment management, and professional nursing care, which play important roles in patients’ postoperative course and enhanced recovery [16]. Our center is one of the largest medical institutions in China for the treatment of renal malignant tumor with venous tumor thrombus. Care for these patients by high-volume providers was associated with greater utilization of cancer surgery, lower Incidence of complications, and better outcomes [17].

This study had the following limitations: 1) The number of patients was relatively small. Studies with larger sample sizes and multi-center data are required for validation. Additionally, this was a retrospective study, and prospective research is required for validation. 2) Most cases reported in this series were Mayo level I and II (61 patients). When a tumor thrombus of Mayo level III was located above the level of the hepatic vein, an open approach was often chosen. However, because of the limited number of Mayo level III patients, the generalizability of the study findings to Mayo level III thrombi was limited. 3) Most of the risk factors analyzed in this study were intraoperative rather than preoperative predictors. There was a lack of preoperative predictors for LCTOA. 4) The incidence of LCTOA was related to the operator’s experience, and there were individual differences. Unfortunately, this study did not objectively evaluate this influencing factor; however, the operators might have had similar experience in such surgery. 5) The choice of surgical instruments was also an important factor affecting the conversion to open surgery. All patients treated with the laparoscopic approach as the first choice were operated using the same manufacturer of surgical instruments, such as ultrasonic cutting devices and bipolar electrocoagulation forceps. However, the frequency of using these surgical instruments was not objectively described and recorded, in our data. Despite these limitations, our study identified the influencing factors for LCTOA and encourages clinicians to pay more attention to these factors preoperatively, to avoid passive change to open surgery intraoperatively. If these influencing factors are present preoperatively, we suggest that the operation could be performed using the open approach or with active LCTOA, which combines the advantages of laparoscopy and an open approach.

## Conclusion

LCTOA was associated with higher median preoperative serum creatinine, longer hospital stay, longer median operation time, and higher median intraoperative hemorrhage volume. However, none of the predictors in our study was an independent risk factor for LCTOA. Perirenal adhesions, organ invasion, and IVC adhesion were the most common causes of LCTOA. Considering the limitations of this study, studies with larger sample sizes are required to validate our conclusions.

## Data Availability

All the data used to support the findings of this study are currently under embargo while the research findings are commercialized. Requests for data 6 months after publication of this article will be considered by the corresponding author at the following e-mail address: malulinpku@163.com (Lulin Ma).

## Abbreviations

LCTOA: Laparoscope conversion to open approach
IVC: Inferior vena cava
RCC: Renal cell carcinoma
RNATT: Radical nephrectomy and tumor thrombectomy
